# Microporous Polyurethane Thin Layer as a Promising Scaffold for Tissue Engineering

**DOI:** 10.3390/polym9070277

**Published:** 2017-07-11

**Authors:** Justyna Kucińska-Lipka, Iga Gubanska, Anna Skwarska

**Affiliations:** 1 Department of Polymers Technology, Faculty of Chemistry, Gdansk University of Technology, Narutowicza St. 11/12, 80-233 Gdansk, Poland; igaguban@pg.gda.pl; 2 Department of Pharmaceutical Technology and Biochemistry, Faculty of Chemistry, Gdansk University of Technology, Narutowicza St. 11/12, 80-233 Gdansk, Poland; annskwar@pg.gda.pl

**Keywords:** polyurethane, scaffold, tissue engineering, SC/PL, TIPS, microporosity, cytotoxic behavior, microbiological studies, porous polyurethane thin layer

## Abstract

The literature describes that the most efficient cell penetration takes place at 200–500 µm depth of the scaffold. Many different scaffold fabrication techniques were described to reach these guidelines. One such technique is solvent casting particulate leaching (SC/PL). The main advantage of this technique is its simplicity and cost efficiency, while its main disadvantage is the scaffold thickness, which is usually not less than 3000 µm. Thus, the scaffold thickness is usually far from the requirements for functional tissue reconstruction. In this paper, we report a successful fabrication of the microporous polyurethane thin layer (MPTL) of 1 mm thick, which was produced using SC/PL technique combined with phase separation (PS). The obtained MPTL was highly porous (82%), had pore size in the range of 65–426 µm and scaffold average pore size was equal to 154 ± 3 µm. Thus, it can be considered a suitable scaffold for tissue engineering purpose, according to the morphology criterion. Polyurethane (PUR) processing into MPTL scaffold caused significant decrease of contact angle from 78 ± 4° to 56 ± 6° and obtained MPTL had suitable hydrophilic characteristic for mammalian cells growth and tissue regeneration. Mechanical properties of MPTL were comparable to the properties of native tissues. As evidenced by biotechnological examination the MPTL were highly biocompatible with no observed apparent toxicity on mouse embryonic NIH 3T3 fibroblast cells. Performed studies indicated that obtained MPTL may be suitable scaffold candidate for soft TE purposes such as blood vessels.

## 1. Introduction

Tissue engineering (TE) has gained more attention in the past decade, owing to its success in enabling tissue regeneration for therapeutic purposes. It is an interdisciplinary field which applies the principles of engineering and life sciences to the development of biological substitutes that restore, maintain or improve tissue function [1] TE aims to produce patient-specific tissue scaffolds in an attempt to skip the limitations of existing clinical treatments for damaged tissues or organs [2]. The main goal of the scaffold is to serve as an adhesion substrate for the cell, provide temporary mechanical support to the newly grown tissue and guide the development of new tissue of appropriate function [3]. Tissue engineering (TE) requires not only suitable biomaterials for scaffold design but also a suitable technique of scaffold fabrication.

Engineered scaffolds should ideally reflect properties of the tissues that are intended to be replaced. Thus, biomaterials used for tissues regeneration, should exhibit complex, mechanically anisotropic behavior optimized for their physiological function [4]. Synthetic polymers are commonly used materials for biomedical applications due to their suitable mechanical properties. Polyurethanes (PURs) are an example of synthetic biomaterials used in the tissue engineering field.

PURs possess segmented structure, which consist of hard and soft segments. Properties of PUR may be tailored by the ratio of raw materials used for their synthesis or by different modifiers and fillers introduced into the PUR structure or matrix [5]. PURs are biomaterials widely used in medical field as wound dressings, artificial heart valves, vascular grafts and tissue scaffolds. They are recognized as biocompatible materials, which do not cause inflammatory reaction of tissues after implantation. Moreover, PURs applied as cellular scaffolds are biodegradable, thus, undergo degradation under conditions of vital organism. This suitable characteristic of PURs is why they are widely developed as scaffolds for tissue engineering applications [6,7,8,9,10,11].

Scaffold morphology, such as porosity (<93%), pore size and their interconnectivity, and scaffold thickness, has major influence on proper tissue regeneration. Scaffold morphology has to be adjusted to the tissue that is to be replaced. Pore size in the range 200–500 µm was recognized as suitable matrices for bone tissue regeneration. On the other hand, skin regeneration occurs when pore sizes are 20–125 µm, liver regenerates properly at scaffolds of pore size in the range 45–150 µm [12] and blood vessels when the pore sizes are up to 38–150 µm [13]. In contrast, microvascular ECM form a connected multicellular lining on scaffolds with pores up to 38 µm [13], so very small pores are necessary, but only to some limit, as small pores may close scaffold structure and cells will be unable to migration deep into the scaffold [12]. Moreover, the regeneration should accelerate the reconstruction of natural ECM collagenous fibers. This is possible only when scaffold is of suitable thickness. Literature reports that proper cells penetration is possible up to 200–500 µm of scaffold depth. In thicker scaffolds, there are no suitable conditions for delivery of nutrients and oxygen to the cells as well as removal of cell metabolite wastes [14,15].

Scaffolds designed for cardiovascular system regeneration have been fabricated in a wide range of forms, such as sponges, meshes or films [4]. All of the mentioned shapes have to have interconnected open-cell structured micropores to enhance the compliance and to increase transmural tissue ingrowths from the surrounding tissues [16]. The micropore-directed fabrication techniques include electrospinning [17], thermally induced phase separation (TIPS) [18] and SC/PL [19,20]. Electrospinning is a unique and versatile process to produce polymeric fibers suitable for vascular graft prostheses [4]. Fibers with a variety of cross sectional shapes and sizes may be produced from different polymers. Electrospun fibers, from polymer solutions and melts, can be obtained in the average diameter range of few nanometers to several micrometers (usually between 50 nm and 5 μm) [6,21]. The weakness of this technique is the difficulty to preserve suitable pore sizes for cellular ingrowth caused by inadequate reproduction of extracellular matrix (ECM), poor reproducibility of the scaffolds and preservation of their restricted architecture [4,22]. TIPS is based on quenching the polymer solution below the solvent’s freezing point (Tk) and inducing liquid–liquid separation. Two phases are formed: a polymer-rich phase and a polymer-poor phase. The polymer-rich phase solidifies, whilst the polymer-poor phase crystallizes. The formed crystals are removed, leaving a highly porous structure (more than 90%) [23,24]. The structure of the obtained scaffold depends on the polymer solution concentration, the quenching temperature and the quenching rate [25]. Solvents application was recognized as the main disadvantage of this technique. SC/PL involves leaching out solid particles from the polymer solution. To the polymer solution, which is usually prepared at a concentration of 5–20% [26], specified diameter particles are added. After solvent evaporation by air-drying, vacuum-drying or freeze-drying, salt particles remain embedded throughout the polymer matrix. After immersion in water, salt particles are leached out, leaving a porous structure. According to Zhu et al. [20], highly porous scaffolds with porosity up to 93% and average pore sizes of up to 500 μm can be obtained with the use of SC/PL technique. The structure of the formed scaffold depends on many factors. The shape and size of pores are directly determined by the shape and dimensions of the leachable particles used [27,28,29]. Salt particles are mainly used, but the use of sugar, ammonium chloride, sucrose, starch particles and gelatine, paraffin microspheres is also known [19,30]. The main advantage of this method is the ease of fabrication without the need of specialized equipment. The disadvantage of this method is, according to Mikos et al. [31], the impossibility to produce thin porous materials below 3000 µm thick. PUR scaffolds prepared by the SC/PL method are mainly used in soft-tissue engineering [32], for example for the repair of coronary arteries [33].

In this study, we report the successful fabrication of novel microporous polyurethane thin layer (MPTL) of 1 mm thick using aliphatic polyurethane synthesized according to the guidelines given in our previous work [5]. Such MPTL has more suitable scaffold architecture for cells penetration in depth of the scaffold [14,15] in comparison to recent reports in this field [31]. To fabricate MPTL scaffold, we used SC/PL and PS technique combined together. The chemical composition and structure of obtained aliphatic MPTL scaffold was studied with the use of Fourier transform infrared spectroscopy (FTIR) and nuclear magnetic resonance spectroscopy (^1^HNMR) [5]. Mechanical properties (tensile strength, elongation at break and Young’s Modulus) as well as contact angle were determined and compared to the native PUR. To characterize the morphology of the MPTL scaffold (porosity, pore size, pores interconnectivity and scaffold thickness) SEM was performed. Biocompatibility of obtained MPTL scaffolds was studied with the use of mouse fibroblast cell line NIH-3T3. Performed studies showed clearly that obtained MPTL scaffold had suitable characteristic for tissue regeneration, and maybe used as a soft tissue scaffold of blood vessels.

## 2. Experimental

### 2.1. Polyurethane Synthesis (PUR)

The synthesis of PUR used in this study was described in details in our previous work [5]. Briefly, PURs were synthesized by standard two-step polymerization procedure [5,34]. In the first step, a prepolymer was obtained with 8% of free isocyanate groups. It was derived from oligomeric α,ω-dihydroxy(ethylene-butylene adipate) (dHEBA) polyester (trade name Polios 55/20; Purinova, Poland) (77 wt %) and linear aliphatic 1,6-hexamethylene diisocyanate (HDI) (Sigma Aldrich, Poznan, Poland) (23 wt %). The prepolymer reaction was carried out at 80 °C for 4 h. In the second step the chain extender 1,4-butanediol (BDO) (POCH, Gliwice, Poland)—was added to obtain PUR of the optimal mechanical properties [5]. Dibutyltin dilaurate (DBTDL), at the amount of 0.5 wt %, was used as a catalyst, commonly engaged in the synthesis of biomedical PUR [17,35]. The reaction mixture was subjected to intensive stirring and then transferred into a mold, set at 80 °C, for 6 h. Then, the samples were left in a drier at 80 °C for 24 h to complete the reaction.

### 2.2. Microporous Polyurethane Thin Layers (MPTL) Fabrication

**Figure 1:**
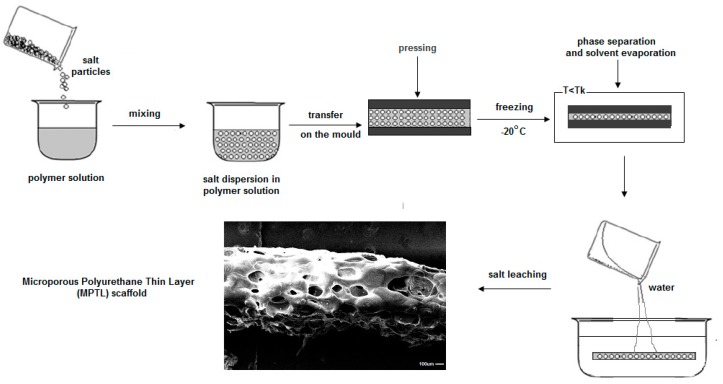
Fabrication steps of MPTL.

To obtain MPTL scaffold SC/PL technique combined with PS was used. PUR was dissolved in 1,4-dioxane (POCH, Gliwice, Poland) at concentration of 20% *wt*/*v*. Then, salt (Culineo, France) of crystals size in the range 0.6–0.4 µm was added to the PUR solution until complete solution saturation occurred. Formulated PUR-salt saturated solution was transferred between the flat stainless steel molds and pressed to reach uniform distribution. Molds were placed at −20 °C overnight to direct the solvent crystallization [22,36,37]. Then MPTL were removed from the mold and immersed in warm (40–50 °C) bidistilled water, where for 7 days the solvent and the sodium particles were washed out. Water was changed twice a day. Finally, samples of MPTL and AA-MPTL were dried at 37 °C for 24 h. The scheme of MPTL fabrication steps is presented in Figure 1.

### 2.3. Fourier Transform Infrared Spectroscopy (FTIR)

The FTIR of the solid PUR and MPTL was performed at FTIR Nicolet 8700 Spectrometer to determine the influence of fabrication procedure on chemical composition of obtained MPTL in comparison to native PUR. The spectral range was from 4000 to 500 cm^−1^, averaging 254 scans per sample with a resolution of 4 cm^−1^.

### 2.4. Proton Nuclear Magnetic Resonance (^1^HNMR)

^1^HNMR spectra of solid native PUR and MPTL were obtained with the use of 500 MHz Varian Spectrometer Unity 500 Plus using deuterated dimethylsulfoxide (DMSO) as PUR solvent and tetramethylsilane as the internal standard of DMSO detectable at 0 ppm. ^1^HNMR was used to determine the possible influence of scaffolds fabrication process on chemical structure of MPTL in comparison to native PUR.

### 2.5. Water Contact Angle

Water contact angle was measured with Reichert Wien optical microscope (35× magnification). Native PUR and MPTL were cut in 2 cm^2^ samples, which surface was purified with *n*-hexane (POCH, Gliwice, Poland) and then dried at 37 °C for 15 min to evaporate hexane before the measurement. To determine contact angle the Sessile Drop Method (SDM) was applied, using a 5 μL of distilled water droplet. For each angle reported, at least 10 measurements on different surface locations were averaged.

### 2.6. Mechanical Properties

Tensile strength (T_Sb_), elongation at break (ε) and Young’s Modulus (E) were studied with the use of Zwick & Roell Z020 testing machine according to PN-EN-ISO 1799:2009. The crosshead speed was of 300 ± 50 mm/min. The measurement was performed at room temperature. Six samples of native PUR and MTPL were studied and the average value of T_Sb_, ε and E is presented in Figure 4.

### 2.7. Scanning Electron Microscopy (SEM)

SEM was performed with the use of SEM Zeiss EVO-40. PUR and MPTL samples were gold-coated before analysis. Image data were imported into Image J software (U.S. National Institutes of Healt, Bethesda, MA, USA) for analysis. The average pore size was obtained by measuring the diameter of 100 pores chosen at randomly throughout the central section of the samples. To perform statistical analysis of pore size we used Shapiro–Wilk test (*p* < 0.05) to determine normal distribution of the data, and to determine the average pore size we used normal Gaussian-Lorentz distribution analysis.

### 2.8. Biocompatibility Studies of MPTL on NIH3T3 Fibroblast Cell Line

#### 2.8.1. Cell Culture

Mouse embryonic fibroblast NIH 3T3 cells were cultured in High Glucose Dulbecco’s modified Eagle’s medium (DMEM HG, Sigma Aldrich, Poznan, Poland) supplemented with 10% fetal bovine serum (FBS) and antibiotics (100 µg/mL each of penicillin and streptomycin) at 37 °C in a humidified atmosphere containing 5% CO_2_.

#### 2.8.2. Indirect MTT Proliferation Assay

The effect of indirect MPTL exposure on NIH 3T3 cell proliferation was determined by 3-(4,5-dimethylthiazol-2-yl)-2,5-diphenyl tetrazolium bromide (MTT) colorimetric assay using 25%, 50% and 100% concentrations of MPTL extract. Briefly, MPTL samples were initially sterilized in 70% ethanol for 30 min and then exposed to UV for 1 h for each side. MPTL samples were subsequently incubated in DMEM HG supplemented with 10% FBS and penicillin/streptomycin for 24 h at 37 °C. The ratio of the total mass of MPTL to the volume of extraction medium was 100 mg/mL. After 24 h, extraction medium was collected, filtered through 0.2-µM filter and used to prepare 25%, 50% and 100% concentrations of MPTL extract. NIH 3T3 cells (2 × 10^4^) were seeded in 24-well plates for 24 h to allow for attachment and then cell culture medium was replaced with MPTL extracts for next 24 h, 48 h and 72 h. DMEM HG supplemented with FBS and antibiotics was used as a non-toxic control. At the end of exposure, 200 µL of MTT solution (4 mg/mL) was added and cells were incubated for 3 h at 37 °C. Then, culture medium was removed and water-insoluble formazan crystals were dissolved in dimethylsulfoxide (DMSO, Sigma Aldrich) and optical density of resulting solutions was measured at 570 nm using iMark Microplate Absorbance Reader (Bio-Rad, Warsaw, Poland). Results were presented as the percentage of cells proliferating after extract exposure relative to control cells cultured in extract-free medium. Obtained data are a mean of two separate experiments wherein each treatment condition was repeated in two wells.

#### 2.8.3. Statistical Analysis

The results are average of two experiments, where each extract was tested twice. One-way ANOVA test followed by Bonferroni test for each comparison was performed using GraphPad Prism 6 software (GraphPad Software Inc., San Diago, CA, USA). *p* values of less than 0.05 were considered as significant (* *p* < 0.05; ** *p* < 0.01; *** *p* < 0.001; **** *p* < 0.0001; ns non-significant).

#### 2.8.4. Analysis of Cellular and Nuclear Morphology

NIH 3T3 cells (2 × 10^4^) were seeded in 24-well plates for 24 h to allow for attachment and then treated with MPTL extracts for next 24 h and 72 h as described above. After exposure, cellular morphology was examined directly from the 24-well plate under Zeiss inverted microscope equipped with AxioCam digital camera (Zeiss, Göttingen, Germany). For analysis of nuclear morphology, NIH 3T3 cells were grown on coverslips for 24 h to allow for attachment and treated with MPTL extracts for 24 h and 72 h. After exposure, coverslips with adherent cells were fixed in 4% paraformaldehyde for 15 min at room temperature, rinsed twice with phosphate buffered saline solution (PBS) and stained for 5 min with 1 µg/mL Hoechst 33342 (Sigma, Poznan, Poland) that binds to DNA. Nuclear morphology was examined under Olympus BX60 fluorescence microscope (Olympus, Tokyo, Japan) equipped with AxioCam digital camera.

#### 2.8.5. Detection of Apoptotic and Necrotic Cell Death by Flow Cytometry

The ability of MPTL to induce cell death was examined using Annexin V-FITC Apoptosis Detection Kit (BD Bioscience, Warsaw, Poland) according to manufacturer’s instructions. Briefly, NIH 3T3 cells (5 × 10^5^) were seeded in 60-mm Petri dishes for 24 h to allow for attachment and then treated with MPTL extracts as described above. After exposure, cells were trypsinized, washed twice in ice-cold PBS and double-stained with Annexin V-FITC and propidium iodide (PI) for 15 min in dark at room temperature. Cells were then analyzed for apoptotic and necrotic cell death using an Accuri C6 flow cytometer using software provided by manufacturer (BD Bioscience).

#### 2.8.6. Flow Cytometry Analysis of Cell Cycle

The effect of MPTL extracts on the cell cycle progression of NIH 3T3 cells was examined using PI/RNase staining buffer (BD Biosceince) according to manufacturer’s instructions. Briefly, NIH 3T3 cells (5 × 10^5^) were seeded in 60-mm Petri dishes for 24 h to allow for attachment and then treated with MPTL extracts as described above. After exposure, cells were trypsinized, washed twice in ice-cold PBS and fixed in ice-cold 80% ethanol at −20 °C overnight. The cells were then centrifuged at 1000 rpm for 5 min, washed twice with ice-cold PBS and stained with propidium iodide in the presence of RNase for 15 min in dark at room temperature. Cells were then analysed for DNA content using an Accuri C6 flow cytometer (BD Bioscience) to determine cell cycle distribution.

## 3. Results

### 3.1. Fourier Transform Infrared Spectroscopy (FTIR)

**Figure 2:**
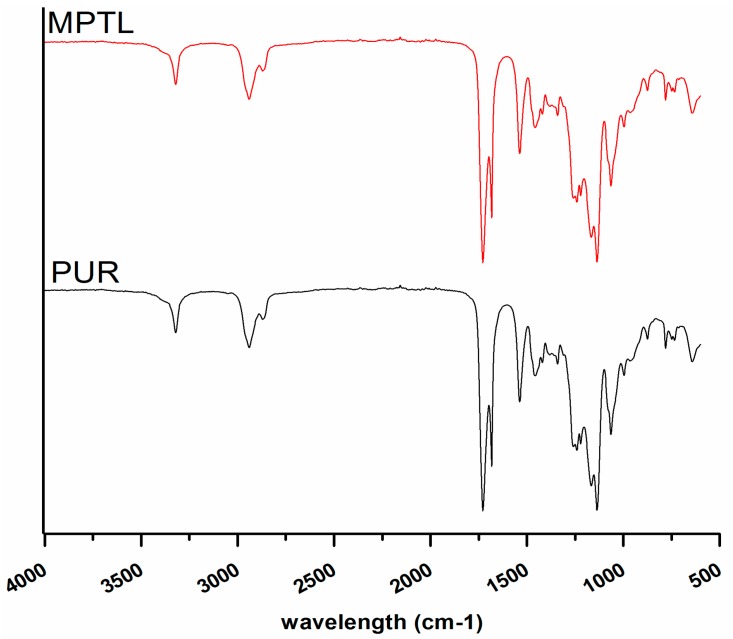
FTIR spectra of PUR and MPTL scaffolds.

Spectra of PUR and MPTL (Figure 2) were similar. The weak absorption peaks, observed at 3319 cm^−1^, were assigned to stretching vibrations of N–H groups present in PUR and MPTL (Table 1). Observed wide baseline of the identified peak of stretching NH groups is related with presence of hydrogen bonds in native PUR and MPTL scaffold. The asymmetric and symmetric stretching vibrations of CH_2_ groups in native PUR and MTPL scaffold composition were noted between 2930 and 2859 cm^−1^. Very strong carbonyl stretching appeared at 1726 cm^−1^ and it is directly related with presence of substantial amount of non-hydrogen bonded or poorly organized hydrogen bonded carbonyl urethane groups in native PUR and fabricated MPTL scaffold. Well ordered and strongly hydrogen bonded urethane groups were recognized in both native PUR and MPTL scaffold by the peak presence at 1655 cm^−1^ [38]. Wavelength of 1527 cm^−1^ was recognized as NH groups deformation vibrations. Signals, indicated in the range of 1451–1314 cm^−1^ concerns planar vibrations of symmetric and asymmetric CH_2_ groups. Peak observed at 1221 cm^−1^ are related with N–C stretching (urethane bonding). Peaks at 1168 cm^−1^, 1138 cm^−1^ for PUR and MPTL, corresponds to the CO–O stretching of ester groups of dHEBA and peaks in the range 969–779 cm^−1^ are related to out of plane bondings vibrations of C–H bending, CH_2_ scissoring; CH_2_ wagging; NH and OH scissoring and wagging. The FTIR analysis confirmed that the performed synthesis lead to obtain polyurethanes and that fabrication process of MPTL scaffold did not influence the chemical composition of resulting scaffold.

### 3.2. Proton Nuclear Magnetic Resonance (^1^HNMR)

**Figure 3:**
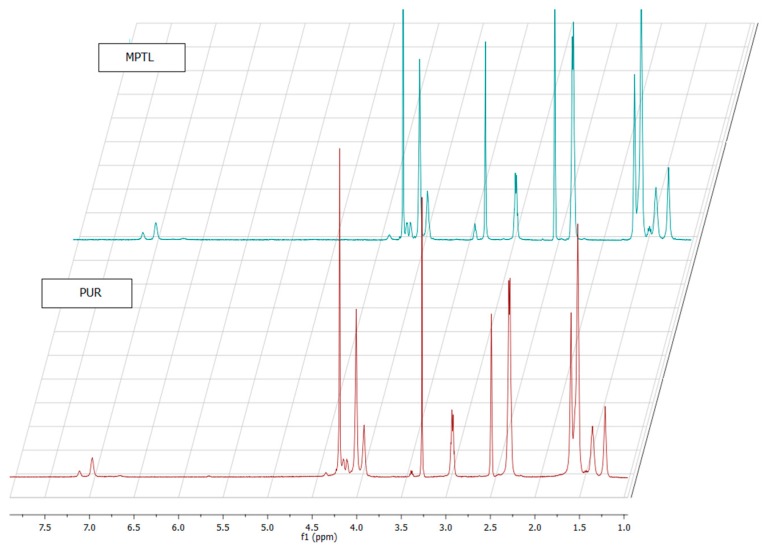
^1^HNMR spectra of obtained PUR and MPTL scaffolds.

The ^1^HNMR spectra of native PUR and MPTL scaffold are similar (Figure 3). Signals of hard and soft segment present in native PUR and MPTL scaffold were confirmed and assignments were presented in Table 2. Polyurethane formation was confirmed by presence of urethane bonding and hydrogen bonding. The processing of native PUR into MPTL scaffold did not affect chemical structure of polyurethane.

### 3.3. Water Contact Angle

Contact angle of native PUR was 78 ± 4° (Table 3). The decrease of contact angle, in comparison to native PUR, was observed for MPTL (56 ± 6°). Fabrication of MPTL scaffold caused decrease of contact angle, which is more suitable for tissue engineering applications. The contact angle of polymeric surface in the range of 45–76° is the most suitable for mammalian cells adhesion and proliferation. Moreover, the ability of polymeric materials, of hydrophilic character, to form hydrogen bonds improves their biocompatibility by solvatation of water molecules, which form at their surface water film that is biologically neutral [39].

### 3.4. Mechanical Properties

#### Tensile Strength (T_Sb_), Elongation at Break (ε) and Young’s Modulus (E)

**Figure 4:**
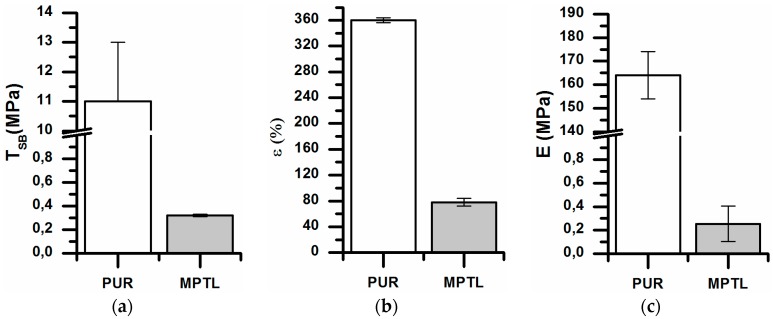
Mechanical properties of native PUR and fabricated MPTL scaffold: (**a**) tensile strength (T_Sb_); (**b**) elongation at break (ε); and (**c**) Young’s Modulus (E).

In Figure 4, mechanical properties of native PUR and fabricated MPTL scaffolds are presented. Native PUR had T_Sb_ of 11 ± 2 MPa, ε of 360 ± 4% and E equal to 164 ± 10 MPa. Fabrication process of MPTL decreased significantly mechanical properties of obtained scaffolds: T_Sb_ was of 0.32 ± 0.01 MPa, ε was of 78 ± 6% and Young’s Modulus was of 0.254 ± 0.15 MPa. Mechanical properties of obtained MPTL were more suitable for soft tissues engineering. For example, artery possesses T_Sb_ in the range of 0.3–0.8 MPa, ε in the range of 50–100% and E in the range of 0.3–1 MPa [40,41]. Moreover, Young’s Modulus of soft tissues usually does not exceed 1 MPa [42].

### 3.5. Scanning Electron Microscopy (SEM)

**Figure 5:**
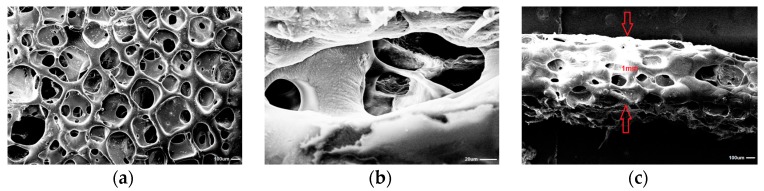
SEM micrographs of MPTL scaffold: (**a**) surface ×100; (**b**) scaffold interior ×10,000; and (**c**) scaffold thickness ×100 times.

Obtained with the use of SC/PL combined with PS technique MPTL was highly porous (82%) (Figure 5). Pore sizes of MPTL were in the range of 65–426 µm with average scaffold pore size of 154 ± 3 µm. The thickness of obtained MPTL was equal to 1 mm. Porosity and pore sizes of obtained MPTL were suitable for regeneration of hard tissues of bones, which regenerates properly at scaffolds of porosity over 60% and pore size of 200–500 µm [12]. Application of selected salt crystal size and pressing step enable to obtain MPTL of 1 mm thick.

### 3.6. Biocompatibility of MTPL on Mouse Embryonic NIH 3T3 Fibroblast Cell Line

**Figure 6:**
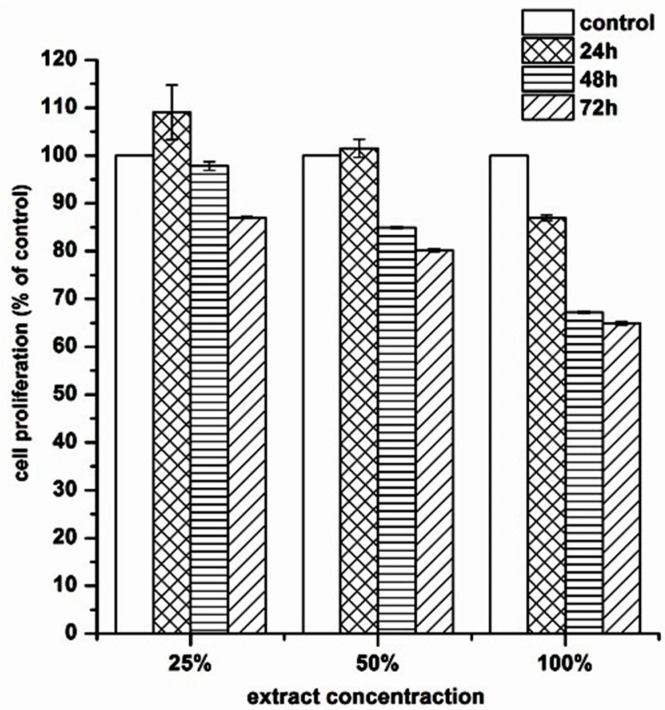
The effect of MPTL extracts on the in vitro growth of mouse embryonic fibroblast NIH 3T3 cells measured using MTT assay. Cell proliferation is represented as a percentage of control cell growth in cultures containing no MPTL extracts. Results are a mean ± SD of two separate experiments wherein each treatment condition was repeated in two wells. * *p* < 0.05; *** *p* < 0.001 vs. control.

The biocompatibility of MTPL on mouse embryonic NIH 3T3 fibroblasts was studied using diverse but complementary approaches that allow monitoring cell proliferation and viability in the presence of different concentrations of MTPL extracts (10%, 25%, 50% and 100%). First, we examined whether MTPL extracts impair NIH 3T3 cell proliferation during 24–72 h exposure (Figure 6). Employing the MTT assay, we showed that MTPL treatment did not substantially affect fibroblast cell growth within first 24-h exposure. For low MTPL extract concentrations (10%, 25% and 50%) the percentage of proliferating cells was similar to that observed for untreated, control cells (95%, 109% and 105%, respectively), whereas for 100% MTPL extract, the percentage of growing cells was 87%. After 48 h and 72 h of cell incubation with 10%, 25% and 50% MTPL extracts, we observed a slight decrease in cell proliferation, which was however still above 80%. Although cell exposure to 100% MTPL extract for 48 h reduced NIH 3T3 cell proliferation by approximately 35%, importantly, such inhibitory effect did not increase during extension of MTPL treatment to 72 h, indicating that majority of cells were still proliferating.

**Figure 7:**
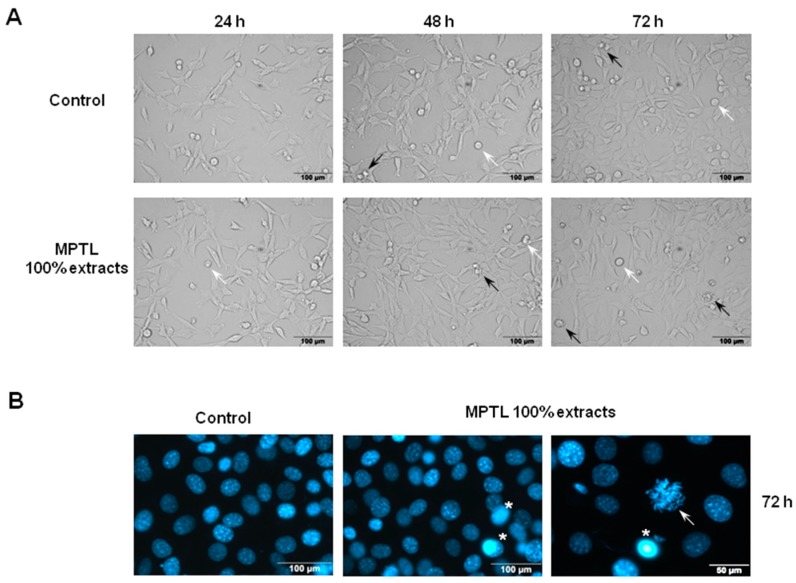
The effect of MPTL extracts on the: cellular (**A**); and nuclear (**B**) morphology of mouse embryonic fibroblast NIH 3T3 cells. (**A**) NIH 3T3 cells were exposed to 100% concentrated MPTL extract for the time indicated and examined under inverted microscope using ×20 objective. Apart from healthy, flattened adherent cells and detached round mitotic cells (white arrows), there were only few shrunken cells with blebbing membrane typical for apoptotic cell death (black arrows); (**B**) NIH 3T3 cells were exposed to 100% concentrated MPTL extract for 72 h and examined under fluorescence microscope using ×40 objective following staining with Hoechst 33342. Shrunken nuclei with intensely stained condensed and pycnotic chromatin were regarded as apoptotic (star). Arrow indicates cell with condensed chromosomes undergoing mitosis.

Next, we examined the effect of MTPL extracts on cellular and nuclear morphology of NIH 3T3 fibroblasts. The images in control and treated cells obtained using bright field inverted microscope show no significant differences (Figure 7A).

Although both control and MTPL-exposed cells show some dead apoptotic cells that are noticeably shrunken with blebbing cell membrane, the majority of cells remain viable even after 72 h of 100% extract exposure. Fluorescence microscopy analysis of nuclear morphology following Hoechst 33342 staining corroborated the above observations. After exposure of NIH 3T3 cells to 100% MTPL extract for 72 h, only occasional apoptotic cells with shrunken and condensed nuclei were observed (Figure 7B). Importantly, as evidenced by the presence of chromosome spreads, cells were able to undergo mitosis in the presence of MTPL.

**Figure 8:**
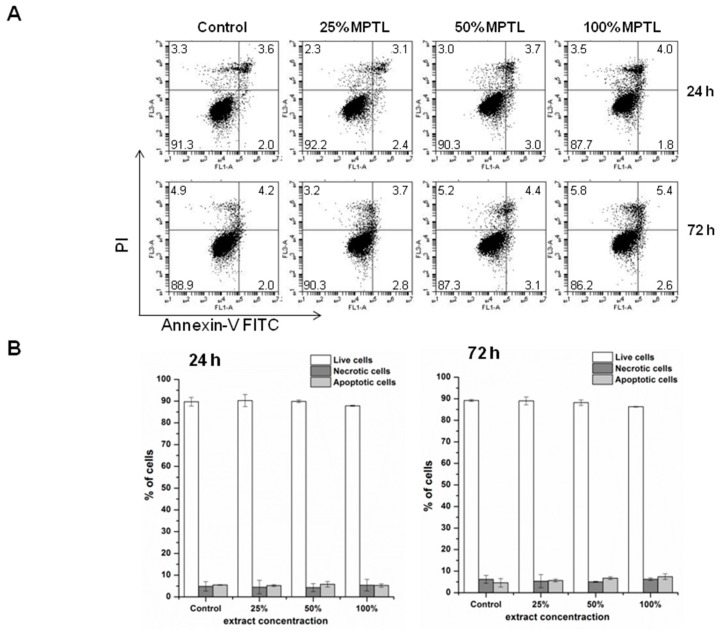
The effect of MPTL extracts on the induction of apoptotic and necrotic cell death of mouse embryonic fibroblast NIH 3T3 cells. Following MPTL extract treatment, cells were double-stained with Annexin V-FITC and propidium iodide (PI) and analyzed by flow cytometry. (**A**) Representative flow cytogram of Annexin V-FITC binding (abscissa) versus PI uptake (ordinate). The lower left quadrant (Annexin V-FITC−/PI−) represents viable cells, the lower right quadrant (Annexin V-FITC+/PI−) was considered as early-stage apoptotic cells, the upper right quadrant (Annexin V-FITC+/PI+) was considered late-stage apoptotic cells, and the upper left quadrant (Annexin V-FITC−/PI+) was considered as necrotic cells; (**B**) Summaries of changes in the percentage of viable, apoptotic (both early and late stage) and necrotic cells, as assessed in the experiments shown in panel A. Results are the mean ± SD of two independent experiments.

Since microscopic examination provided qualitative description of the effect of MTPL on NIH 3T3 functioning, next we performed quantitative flow cytometry analysis of NIH 3T3 viability using dual Annexin V-FITC and propidium iodide staining (Figure 8).

Anticoagulant protein Annexin V conjugated with fluorescein isothiocyanate (FITC) binds to phosphatidylserine residues that are exposed on cell membrane during early and late stages of programmed cell death by apoptosis [43]. Counter staining cells with PI allows for contemporary identification of second type of cell death within treated population, e.g., necrosis. Necrotic cells due to the compromised membrane will incorporate PI that is normally excluded by viable, healthy cells. Of note, cells at the late stage of apoptosis, next to Annexin V-FITC also accumulate PI. Flow cytometry analysis revealed no significant differences between control and MTPL-treated cells (Figure 8A,B). Majority of cells (over 85%) remained viable (Annexin V-FITC negative, PI negative) regardless the concentration of MTPL extracts or time of exposure. The level of cells that were dying by apoptosis or necrosis collectively did not exceed 15% even at 100% MTPL extract exposure for 72 h. These observations clearly indicate that although 100% concentrated extract of MTPL slightly inhibits cell proliferation as shown by MTT assay, it is not toxic to NIH 3T3 fibroblasts and does not induce cell death by apoptosis or necrosis. To confirm these observations we also determined the cell cycle phenotype of NIH 3T3 cells by flow cytometry.

**Figure 9:**
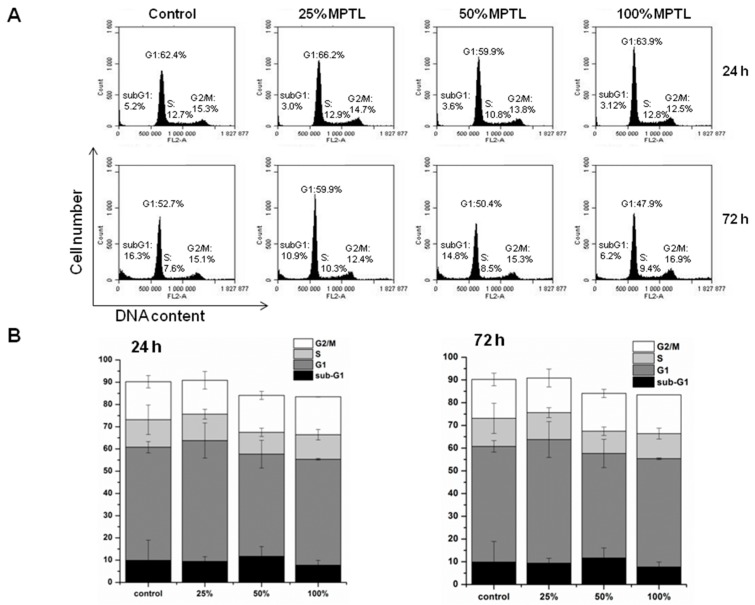
The effect of MPTL extracts on mouse embryonic NIH 3T3 fibroblasts cell cycle distribution. Following MPTL extract treatment, cells were stained with propidium iodide and percentages of cells in the subG1, G1, S and G2/M phases were determined by flow cytometry. (**A**) Representative DNA content histograms. Cells in G2/M phases have double the DNA content than those in G1 phase. S-phase cells have DNA content lying between that for G1 and G2/M phases. The sub-G1 region represents apoptotic cells with fragmented DNA; (**B**) Summaries of changes in the percentage of cells in different cell cycle phases, as assessed in the experiments shown in (**A**). Results are the mean ± SD of two independent experiments.

As shown in Figure 9A,B, MTPL treatment, regardless extract concentration and time of exposure, did not significantly increase the apoptotic sub-G1 population of cells with lower DNA content resulting from lethal DNA fragmentation.

Moreover, even extended exposure (72 h) to 100% concentrated MTPL extract did not affect distribution of NIH 3T3 cells in G1, S and G2/M phases. These results indicate that MTPL neither interfered with NIH 3T3 cell cycle progression nor induced cell death.

## 4. Discussion

The loss or failure of an organ or tissue is one of the most frequent, devastating, and costly problems in healthcare. Current treatment modalities include transplantation of organs, surgical reconstruction, use of mechanical devices, or supplementation of metabolic products. A new field, tissue engineering, applies the principles and methods of engineering, material science, cell and molecular biology toward the development of viable substitutes which restore, maintain, or improve the function of human tissues [44].

To achieve this goal a suitable scaffold has to be designed. Such scaffolds have to have suitable macro- and microstructure to promote cell proliferation. Such characteristic possess obtained in this study MPTL. The chemical composition and structure of MPTL, studied with the use of FTIR and ^1^HNMR, was maintained in comparison to native PUR. On the other hand, processing of PUR into the MPTL significantly influenced its hydrophilic and mechanical properties. Contact angle of MPTL (56 ± 6°) substantially decreased in comparison to native PUR (78 ± 4°). Noted 22° decrease of contact angle caused that obtained MPTL was hydrophilic and suitable for tissues regeneration. Literature reports that the contact angle, of polymeric surface, in the range of 45–76° is the most suitable for mammalian cells adhesion and proliferation. Moreover, the ability of hydrophilic-polymeric materials to form hydrogen bonds improves their biocompatibility by solvatation of water molecules, which form at their surface biologically neutral water film [39]. Mechanical properties of MPTL decreased and reached suitable values for soft tissue scaffolds (T_Sb_ = 0.32 ± 0.01 MPa, ε = 78 ± 6% and E = 0.254 ± 0.15 MPa). For example, aorta possess T_Sb_ in the range of 0.3–0.8 MPa, ε between 50% and 100% and Young’s Modulus in the range of 0.3–1 MPa [40,41]. Moreover, Young’s Modulus of soft tissues usually does not exceed 1 MPa [42]. SEM analysis confirmed that used SC/PL combined with PS technique provided highly porous structure of obtained MPTL. Porosity of MPTL was of 82%, pore size was in the range of 65–426 µm, and an average MPTL pore size was of 154 ± 3 µm. Recognized morphology of obtained MPTL suitable for bone tissue regeneration, which regenerates properly at scaffolds pore sizes between 200 and 500 µm and porosity over 60% [12]. Used combination of SC/PL and PS techniques provided more suitable scaffold thickness for cells ingrowth in depth of the scaffold [14,15]. The MTT assay revealed that MTPL did not substantially affect fibroblast cell growth within first 24-hour exposure. The low MTPL extract concentrations (10%, 25% and 50%) did not significantly affect cells proliferation, which was similar to that observed for untreated, control cells (95%, 109% and 105%, respectively), whereas for 100% MTPL extract, the percentage of growing cells was 87%. After 48 and 72 h of cell incubation with 10%, 25% and 50% MTPL extracts, a slight decrease in cell proliferation was observed, which was however still above 80%. Although cell exposure to 100% MTPL extract for 48 h reduced NIH 3T3 cell proliferation by approximately 35%, importantly, such inhibitory effect did not increase during extension of MTPL treatment to 72 h indicating that majority of cells was still proliferating. The cells morphology studied by bright field inverted microscope did not indicated significant differences in comparison to controls. The majority of cells remained viable even after 72 h of 100% extract exposure. Fluorescence microscopy analysis of nuclear morphology following Hoechst 33342 staining confirmed above mentioned observations. After exposure of NIH 3T3 cells to 100% MTPL extract for 72 h, only occasional apoptotic cells with shrunken and condensed nuclei were observed. Importantly, as evidenced by the presence of chromosome spreads, cells were able to undergo mitosis in the presence of MTPL. Performed quantitative flow cytometry analysis of NIH 3T3 viability using dual Annexin V-FITC and propidium iodide staining revealed no significant differences between control and MTPL-treated cells. Majority of cells (over 85%) remained viable (Annexin V-FITC negative, PI negative) regardless the concentration of MTPL extracts or time of exposure. The level of cells that were dying by apoptosis or necrosis collectively did not exceed 15% even at 100% MTPL extract exposure for 72 h. These observations clearly indicate that although 100% concentrated extract of MTPL slightly inhibits cell proliferation as shown by MTT assay, it is not toxic to NIH 3T3 fibroblasts and does not induce cell death by apoptosis or necrosis. These observations were confirmed by determination of the cell cycle phenotype of NIH 3T3 cells by flow cytometry. This study indicated that the MTPL treatment, regardless to extract concentration and time of exposure, did not significantly increase the apoptotic sub-G1 population of cells with lower DNA content resulting from lethal DNA fragmentation.

Moreover, even extended exposure (72 h) to 100% concentrated MTPL extract did not affect distribution of NIH 3T3 cells in G1, S and G2/M phases. These results indicate that MTPL neither interfered with NIH 3T3 cell cycle progression nor induced cell death. Summarizing, obtained MPTL scaffold was highly biocompatible, thus it can be concluded that optimal conditions to cell proliferation and growth were provided by fabricated MPTL. Performed studies showed that MPTL fabricated by using SC/PL combined with PS technique meet the physicochemical, mechanical and biological requirements of scaffolds dedicated to the soft tissue engineering such as blood vessels.

## 5. Conclusions

In this study, we successfully obtained novel MPTL of 1 mm thickness, which is more suitable for cellular ingrowth in depth of the scaffold according to the requirements [14,15]. Such MPTL maintained its chemical composition and structure after processing with the SC/PL combined with PS technique, which was confirmed by FTIR and ^1^HNMR study. Optimal processing conditions provided suitable morphology of obtained MPTL for tissues regeneration. MPTL porosity achieved 82% and the average pore size was 154 ± 3 µm. Thus, MPTL may be a good scaffold candidate for vascular tissue regeneration [12]. Significant improvement of hydrophilic and mechanical properties (T_Sb_ = 0.32 ± 0.01 MPa, ε = 78 ± 6%, and E = 0.254 ± 0.15 MPa) of MPTL was obtained, which meet the requirements of soft tissue regeneration such as blood vessels and aorta. Obtained MPTL was highly biocompatible, as confirmed by diverse but complementary approaches allowing the monitoring of cell proliferation and viability in the presence of different concentrations of MTPL extracts (10%, 25%, 50% and 100%). It seems that optimal conditions for cells proliferation and growth were provided by fabricated MPTL. Performed studies showed that obtained MPTL can meet the requirements of tissue scaffolds used for blood vessels regeneration and may be applied as cellular scaffolds in this field of regenerative medicine.

## Figures and Tables

**Table 1:** Spectral data * and band assignments of FTIR analysis presented in Figure 2.

Native PUR and MPTL Scaffold	
Wavelength (cm^−1^)	Assignments
3319 w	N–H stretching, (urethane bonding)
2930 w, 2859 w	CH_3_; CH_2_ (aliphatic) asymmetric and symmetric stretching bonding
1726 vs, 1655 w	C=O stretching of non-hydrogen bonded urethane groups and well ordered and strongly hydrogen bonded urethane groups respectively
1527 m	deformation bond N–H
1451 w, 1420 vw, 1354 vw, 1314 s	planar bonds of symmetric and asymmetric CH_2_
1221 s	N–C stretching (urethane bonding)
1168 s, 1138 s	CO–O stretching of ester
1080 m, 1036 m	C–O stretching of urethane groups
969 vw, 943 vw, 871 vw, 779 w	Out of plane bondings of C–H (bending), CH_2_ scissoring; CH_2_ wagging; NH and OH scissoring and wagging

vw—very weak, w—weak, m—medium, s—strong, vs—very strong.

**Table 2:** Chemical shifts assignments of 1HNMR analysis presented in Figure 3.

	Native PUR and MPTL Scaffold
Chemical Shift (ppm)	Assignments
1.0–1.25	protons from –OH groups in BDO chain extender, which ends the PEUs chains;
1.26–1.40	protons from –NH– groups from urethane bonding: –NH–(C=O)–O–
1.5–1.75	protons from aliphatic –CH_2_– groups without neighborhood of functional groups
2.0–2.40	protons from aliphatic –CH_2_– groups in macrodiol, connected with carbonyl side of ester group; –(C=O)–O–; aliphatic –CH_2_– groups connected with BDO chain extender
2.75–3.0	protons from aliphatic –CH_2_– connected with urethane bonding from –NH– side; –NH–(C=O)–O–
2.50	Solvent—DMSO
3.25–3.45	aliphatic –CH_2_– groups in macrodiol, connected with ester group from the oxygen side; –O–(C=O)–
3.75–4.45	aliphatic –CH_2_– groups connected with urethane group from the oxygen side; –O–(C=O)–NH and protons
6.9–7.15	Signals of hydrogen bonding present in the PUR and MPTL structure

**Table 3:** Water contact angle of native PUR and MPTL scaffold.

Sample	Water Contact Angle (Average ± SD°)
PEU	78 ± 4
MPTL	56 ± 6
